# Ionizable Amino Lipids Distribution and Effects on DSPC/Cholesterol Membranes: Implications for Lipid Nanoparticle Structure

**DOI:** 10.1021/acs.jpcb.3c01296

**Published:** 2023-07-27

**Authors:** Sepehr Dehghani-Ghahnaviyeh, Michael Smith, Yan Xia, Athanasios Dousis, Alan Grossfield, Sreyoshi Sur

**Affiliations:** † Moderna, Inc., Cambridge, Massachusetts 02139, United States; ‡ Theoretical and Computational Biophysics Group, NIH Center for Macromolecular Modeling and Bioinformatics, Beckman Institute for Advanced Science and Technology, Department of Biochemistry, and Center for Biophysics and Quantitative Biology, University of Illinois at Urbana−Champaign, Urbana, Illinois 61820, United States; § Department of Biochemistry and Biophysics, University of Rochester Medical Center, Rochester, New York 14642, United States

## Abstract

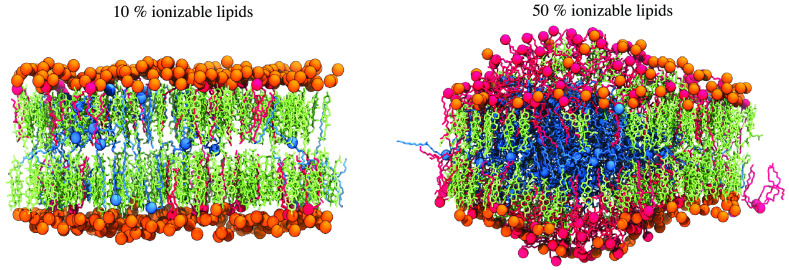

Lipid nanoparticles (LNPs) containing ionizable aminolipids are among the leading platforms for the successful delivery of nucleic-acid-based therapeutics, including messenger RNA (mRNA). The two recently FDA-approved COVID-19 vaccines developed by Moderna and Pfizer/BioNTech belong to this category. Ionizable aminolipids, cholesterol, and DSPC lipids are among the key components of such formulations, crucially modulating physicochemical properties of these formulations and, consequently, the potency of these therapeutics. Despite the importance of these components, the distribution of these molecules in LNPs containing mRNA is not clear. In this study, we used all-atom molecular dynamics (MD) simulations to investigate the distribution and effects of the Lipid-5 (apparent p*K*_a_ of the lipid nanoparticle = 6.56), a rationally designed and previously reported ionizable aminolipid by Moderna, on lipid bilayers [*Mol. Ther.***2018**, *26*, 1509–1519]. The simulations were conducted with half of the aminolipids charged and half neutral approximately to the expected ionization in the microenvironment of the LNP surface. In all five simulated systems in this work, the cholesterol content was kept constant, whereas the DSPC and Lipid-5 concentrations were changed systematically. We found that at higher concentrations of the ionizable aminolipids, the neutral aminolipids form a disordered aggregate in the membrane interior that preferentially includes cholesterol. The rules underlying the lipid redistribution could be used to rationally choose lipids to optimize the LNP function.

## 1 Introduction

Recent biopharmaceutical advances in messenger RNA (mRNA)-based therapeutics have created new opportunities to employ mRNA as a drug.^1−5^ Engineered mRNAs are delivered as cargo to target cells, causing them to express proteins which can be used in treatment of genetic diseases, for gene editing, or as vaccines (e.g., SARS-COV-2 mRNA-based vaccines).^6−8^ The successful clinical application of mRNA-based therapeutics requires efficient intracellular delivery of mRNA to the cytoplasm of target cells, enhancing the expression of desired proteins.^9^ In vivo delivery of naked mRNA results in poor cellular internalization, rapid degradation, and fast renal clearance.^10^ Lipid nanoparticles (LNPs) are nonviral vectors extensively employed in therapeutic applications of mRNA delivery.^11−14^ LNPs have been developed to protectively encapsulate and deliver mRNA to target cells.^13,15,16^

LNPs are complex structures, generally consisting of (1) a phospholipid, providing structure to LNP, (2) cholesterol, enhancing LNP stability, and (3) an ionizable lipid, capturing the mRNA (4) and PEG lipids, prolonging the stability in the bloodstream. The presence of ionizable lipids facilitates cellular uptake and endosomal escape of mRNA to the cytoplasm.^13,17^ Ionizable aminolipids are titratable molecules that can be charged or neutral at different pH values.^18−20^ Encapsulation of the negatively charged mRNA in LNPs is achieved by generating a mRNA–lipid mixture at acidic pH, where the ionizable aminolipids are positively charged and favor charge-driven interactions with mRNA.^15,19^ After the encapsulation process, the pH is raised above the LNP’s p*K*_a_ value, resulting in a near-neutral surface charge, which is desirable for clinical applications.^15,19,21^ Investigations of the LNP assembly process have shown that changing the buffer pH alters the protonation state of the aminolipids and plays a key role in mRNA encapsulation.^22^ Cryo-transmission electron microscopy (cryo-TEM) images revealed that the chemical structure and the proportion of the ionizable aminolipids extensively affect the size and morphology of the LNPs.^23,24^ In this work, we have used molecular dynamics simulations to understand the relationship between the amount of aminolipids in the LNP and the LNP surface characteristics.

**Figure 1:**
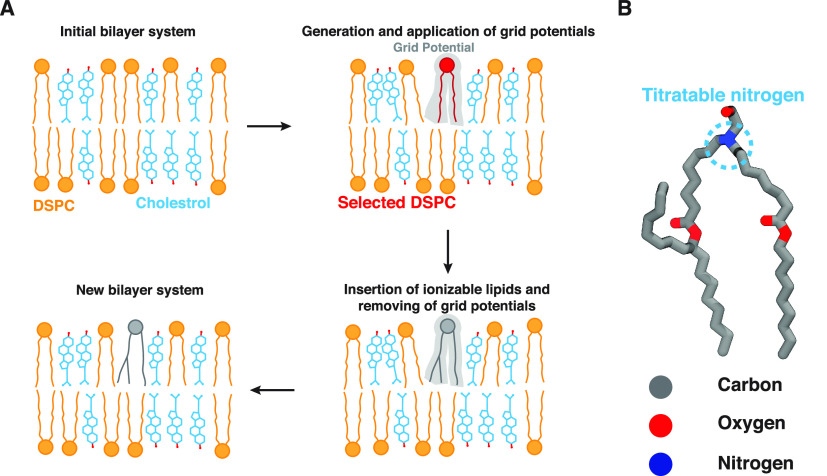
Lipid-5 insertion algorithm. (A) Different steps involved in the insertion algorithm. The first step involves an initial equilibrated bilayer system as input to the algorithm. Here, the initial system consists of DSPC and cholesterol lipids, shown in blue and orange, respectively. The headgroups of DSPC are shown in orange circles, where the hydroxyl group of cholesterol is represented in red dots. The first step is followed by random selection of a predetermined number of DSPC molecules (shown in red). A series of repulsive grid potentials (shown in faded gray) are generated at the place of selected DSPCs, generating enough space to insert ionizable lipids. Lipid-5 (shown in gray) is inserted into the bilayer followed by gradual removal of the grid potentials. Finally we generate a new bilayer containing the desired proportion of the ionizable lipid. (B) Lipid-5 molecular structure. The nitrogen in Lipid-5 is titratable and can change its protonation depending on the buffer pH. Carbon, oxygen, and nitrogen atoms are shown in gray, red, and blue, respectively.

Due to the importance of ionizable aminolipids in the assembly process and final properties of LNP, advances in mRNA therapeutics require a major focus on optimizing the chemical structure of ionizable aminolipids and the proportion of different lipids used in LNP assembly.^19,24,25^ With a new series of novel ionizable lipids, Moderna has shown in previous work the improvement of mRNA endosomal escape and sustained safety for LNP-based delivery of mRNA.^14,19,23,24^ In addition, employing the novel ionizable aminolipids in LNP construction and assembly leads to improved biodegradability and maintaining immune titers for mRNA therapeutics.^14,19^ Here, we focus on the novel ionizable aminolipid identified as Lipid-5 in our previous study.^19^ Lipid-5 contains a titratable nitrogen atom connected to two saturated and esterified branched and unbranched chains (see Figure 1B).^19^ Lipid-5 is a pH-sensitive molecule (apparent p*K*_a_ of LNP = 6.56), whose protonation and deprotonation are necessary for efficient encapsulation and endosomal escape of mRNA.^19^ Lipid-5 is an ionizable aminolipid extensively used for delivery of therapeutic mRNAs to the liver.^19^ This lipid is an integral part of the LNPs that deliver mRNAs to treat methylmalonic acidemia, arginase deficient liver disorder, Fabry disease, galactose sensitivity, and hemophilia in mouse models.^26−30^ Although the critical role of Lipid-5 in LNP structure and properties has been a point of focus of several investigations,^24^ molecular interactions between Lipid-5 and other LNP’s components and the specific role of this ionizable aminolipid on the membrane surface are not clear.

Molecular dynamics (MD) simulation is a technique used to provide insights, with atomistic resolution, on the structure and dynamics of a wide variety of biomolecular systems of pharmaceutical interest, including biological membranes and lipid-based drug delivery systems.^31,32^ Due to the large computing resources required for all-atom molecular dynamics simulations of large biomolecular systems, the lipid bilayer model is often used as a section from LNP surfaces in MD simulations. The bilayer model can be complex and composed of various lipid molecules, e.g., phospholipids, sphingolipids, and sterols.^33^ Previous simulations of a system containing the ionizable aminolipid DLinKC2-DMA (KC2), a phospholipid, cholesterol, a poly(ethylene glycol) (PEG) lipid, and siRNA in conjunction with cryo-TEM and H NMR revealed that the core of the LNP consists of a mixture of ionizable aminolipids, cholesterol, and phospholipids interacting with RNA.^34^ Additionally, this study revealed that upon increasing the pH during the LNP formulation process, KC2 confinement at the hydrophobic core of the bilayer could result in rearrangement of the internal structure of the LNP.^35^ An all-atom MD simulation of a membrane bilayer containing DLin-MC3-DMA (MC3) and DOPC or DOPE suggested that DOPE-MC3 intermolecular interactions have higher affinity than DOPC-MC3 interactions. They also showed that MC3 molecules segregate at the surface of the membrane and that lipid molecules diffuse more slowly in the MC3-DOPE membrane system.^36^ Another study by Park et al. developed a protocol to build lipid bilayers containing particular ionizable lipids and PEG lipids in addition to other lipids already available in CHARMM-GUI web server.^37^ This study investigates the effects of PEG lipids on the lipid bilayer system using molecular dynamics simulations.^37^

Despite several MD simulation-based investigations of other ionizable aminolipids, the impacts of changing the molar ratio of aminolipid-to-phospholipid in a lipid bilayer system has not been characterized previously.^35−37^ We also developed a protocol in this work to incorporate any novel ionizable lipid into the lipid bilayer system as a starting structure for MD simulations. The present work is also the first time we investigate the impact of Lipid-5 in a lipid bilayer system. Here, we use all-atom MD simulations to study the role of Lipid-5 in shaping the characteristics of five ternary membrane bilayers containing different compositions of Lipid-5, phospholipids, and cholesterol.

Our simulations predict that protonated Lipid-5 molecules are largely found on the surface, while the neutral molecules partition to the hydrophobic core of the bilayer. Several properties of membrane bilayer systems were monitored and characterized throughout the simulations, suggesting that populating the bilayer with Lipid-5 (1) induces aggregation of neutral and protonated ionizable aminolipids in the hydrophobic core and on the surface of the bilayer, respectively, (2) increases membrane fluidity, and (3) increases the partitioning of cholesterol into the hydrophobic core of the bilayer. We hypothesized that the core of the LNP is composed of mostly neutral ionizable aminolipids and cholesterol for ethanolamine ionizable aminolipids. The finding of this investigation can be employed in designing an appropriate lipid composition for LNPs in mRNA therapeutics to optimize LNP interactions with the cell membrane, endocytosis, endosomal escape, and fluidity of the LNP surface.

## 2 Methods

### 2.1 Lipid-5 Insertion Algorithm

The ionizable aminolipid used in this study, Lipid-5, does not exist in CHARMM-GUI’s^38,39^ lipid libraries, which led us to develop an insertion algorithm to incorporate Lipid-5 in our bilayer systems (Figure 1A). The initial development of this algorithm was inspired by the Membrane Mixer module of VMD, in which a new workflow was proposed for fast lipid mixing in MD-modeled bilayers.^40^

The algorithm developed here applies a volumetric repulsive potential, known as grid potential, to reduce steric clashes between Lipid-5 and other components of the simulation box and contains the following steps:

(1) We start by constructing an initial equilibrated bilayer, without Lipid-5, employing available tools, e.g., CHARMM-GUI or Optimal Membrane Generator (OMG), a package of LOOS (Figure 1A, top left corner).^38,39,41^
(2) The algorithm selects a number of lipid molecules present in the initial structure and to be replaced by ionizable aminolipids. This selection is random, and the number of selected lipid molecules in each leaflet depends on the final desired proportion of ionizable aminolipids, Lipid-5 in this case, specified by the user. Furthermore, in the case of heterogeneous lipid bilayers users can specify which lipid type should be selected and replaced by the insertion algorithm (Figure 1A, top right corner).
(3) Repulsive grid potentials at the place of the selected lipid molecules are generated from the previous step. The grid potentials resemble the cylindrical shape of the Lipid-5 molecule with the tails pointing toward the hydrophobic core of the membrane and the headgroup exposed to water (Figure 1A, top right corner).
(4) We run MD with the grid potentials to make room for the soon to be inserted Lipid-5 molecules into the bilayer. We mention that the grid potentials are not applied to the selected lipid molecules in step 2, and they just shape the environment surrounding each selected lipid as if there is a Lipid-5 molecule located there (Figure 1A, top right corner).
(5) The selected lipid molecules are replaced with Lipid-5 (Figure 1A, bottom right corner).
(6) The grid potential is removed gradually, making sure the lipid bilayer has sufficient amount of packing. In order to achieve this goal, the algorithm linearly decreases the force constant of the grid potential in eight steps; the first and last steps are simulated with the original force constant employed in step 4 and no grid potential, respectively (Figure 1A, bottom left corner). Furthermore, this step contains a procedure, which was developed previously, to remove any possible ring piercing of the cholesterol molecules.^40,42^

The algorithm is implemented as a tcl script in VMD and takes advantage of several MD simulation/analysis packages.^42^ The construction of the new system with ionizable aminolipids as well as the insertion process itself is conducted with VMD and AmberTools ver. 20.^42,43^ The grid potentials are generated using the molecular dynamics flexible fitting (MDFF) plugin of VMD with resolution and spacing of 6 and 0.8 Å, respectively.^44^

### 2.2 Construction and Parametrization of the Ionizable Aminolipid, Lipid-5

Heptadecan-9-yl 8-((2-hydroxyethyl)(8-(nonyloxy)-8-oxooctyl)amino)octanoate, referred to here as Lipid-5, contains a nitrogen atom that is connected to two saturated chains (Figure 1B). The nitrogen atom of Lipid-5 is titratable (apparent p*K*_a_ of LNP = 6.56) and can be protonated or neutral, causing the net charge of Lipid-5 to be +1 or 0, respectively.^19^ This implies Lipid-5 will be gaining and losing a proton quickly in solution. Because molecular dynamics simulations cannot emulate bond formation or bond cleavage, we consider a racemic mixture of Lipid-5 with the titratable nitrogen as the chiral center in our system. Hence, we constructed three different structures for Lipid-5 using Schrödinger LigPrep ver. 2021-4 and OpenBabel ver. 3.1.0.^45,46^ We then parametrized each ionizable aminolipid structure employing Ambertools ver. 20 and Generalized Amber Force Field (GAFF2).^43,47,48^

### 2.3 System Preparation

The initial membrane bilayer system consisting of 1,2-distearoyl-*sn*-glycero-3-phosphocholine (DSPC) and cholesterol was constructed, solvated, and ionized with 150 mM NaCl, employing CHARMM-GUI.^39,49^ Initially, the lipid bilayer was equilibrated in two different steps: (1) restraining lipid headgroups in the direction of the membrane normal for 1.125 ns and (2) 1 ns equilibration without any restraints. The relaxed lipid bilayer system was then passed to the insertion algorithm for incorporating different proportions of Lipid-5.

Five different systems were constructed from the original bilayer system with Lipid-5 occupying 10, 20, 30, 40, and 50% of the population of the lipid molecules and replicated three times using the Insertion algorithm discussed in Section 2.1. In order to avoid any bias generated by the initial configuration of lipids, each replica was constructed independently, using the insertion algorithm. In all the prepared systems, a certain number of DSPC molecules were replaced by Lipid-5 (based on the desired proportion of Lipid-5 in the final bilayer structure), keeping the cholesterol concentration constant (40%). At step 4 of the insertion algorithm, each system was equilibrated for 10 ps. Removing the grid potentials (step 6) was conducted in 50 ps. After the incorporation of Lipid-5 into bilayers, each system was run for 3 μs without any biasing potentials (Table S1).

### 2.4 Simulation Protocol

The MD simulations described here were conducted employing the NAMD3 software package.^50,51^ The AMBER Lipid 17 parameters were employed for DSPC and cholesterol molecules. We used the sodium and chloride ions parameters from Li-Merz OPC-HFE parameters for monovalent ions, and we used the TIP3P water model.^47,48,52,53^ We used GAFF to parametrize Lipid-5, and we parametrized both the neutral and the charged ionizable aminolipid separately, using the antechamber tool.^48^ A 9 Å cutoff was used for short-range nonbonded interactions. Long-range electrostatic interactions were calculated using the particle mesh Ewald (PME) method^54^ with a grid density of 1 Å^–3^ and a spline interpolation order of 6. All bonds involving hydrogen atoms were kept rigid employing the SETTLE algorithm.^55^ The temperature was set to 310 K by using a Langevin thermostat with a damping coefficient of 1.0 ps^–1^. The pressure was Langevin piston barostat (period: 50 fs; decay: 25 fs).^56,57^ All of the simulations were run in a flexible cell, allowing the dimensions of the periodic cell to change independently while keeping the aspect ratio in the *x–y* plane fixed. The simulation box size was initially set to 125 Å × 125 Å × 90 Å. The simulation time step was set to 2 fs. Lennard-Jones and PME forces were updated every and every other timesteps, respectively. During the system construction, biased simulations with grid potentials were performed using the grid-steered MD module in NAMD.^58^ MD trajectories were visualized with VMD.^42^ Each system (with a certain lipid composition) was independently constructed three times, using our developed technique to avoid any bias from the initial configuration of lipid molecules. Each membrane bilayer was then simulated for 3 μs, generating 9 μs of sampling for each lipid composition and cumulative simulation time of 45 μs for all the systems.

### 2.5 Analysis

All the analyses are performed over the last 1 μs of each trajectory, using VMD ver. 1.9.4, LOOS ver 3.0.0, and MDAnalysis ver. 1.1.1.^42,59,60^ The error bars represented in some of the analyses are standard deviations between the replicates of each system. The reported values and distributions for scaled mass densities, lipid orientation angles, lipid order parameters, cholesterol partition coefficients, and total area of the bilayers are averaged over all three replicates, where the lipid aggregation analyses were performed separately for each replicate.

#### 2.5.1 Scaled Mass Density

The scaled mass density along the membrane normal was calculated for the titratable nitrogen atoms for protonated and neutral Lipid-5. In this calculation, the simulation box is divided into different slices in *z*, 1.4 Å apart from each other. The population of nitrogen atoms in each slice is counted, and the highest value in each distribution is scaled to one. We used the MDAnalysis^60^ and LOOS^41^ to perform this calculation.

#### 2.5.2 Crossing Angles

We computed crossing angles between adjoining ionizable aminolipid’s unbranched tails to quantify the internal structure of the membrane surface as shown in Figure 3C. We used the cross_dist tool in LOOS to calculate the probability distribution of the cosine of the crossing angles with a 10 Å cutoff and 25 bins.^41^

#### 2.5.3 Lateral Radial Distribution Function

We computed the two-dimensional lateral radial distribution function in the membrane plane for the centroids of the protonated ionizable lipids with a resolution of 1 Å, using the LOOS tool xy_rdf.^41^

#### 2.5.4 Orientation Angle and Modified Order Parameter

The orientation angle of Lipid-5 molecules, θ, is defined as an angle between a vector starting from the center of mass of two oxygen atoms on the tail of Lipid-5 and passing through the nitrogen atom located at the head with respect to the membrane normal. DSPC orientation angle is defined as the angle between a vector starting from the center of mass of the tail and passing through the headgroup center of mass of the lipid molecule with respect to membrane normal.

The modified order parameter for the whole Lipid-5 as well as DSPC molecule is calculated using the following equation:
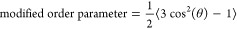
1where θ is the orientation angle.

#### 2.5.5 Lipid-5 Aggregation at the Core and on the Surface of the Bilayer

In order to capture any aggregation of protonated and neutral Lipid-5 at the core and on the surface of the bilayer, respectively, we obtained spatial distributions of the titratable nitrogen atoms of the Lipid-5 molecules. Projection of the coordinates of neutral Lipid-5 into the *x–z* plane was employed to quantify Lipid-5 aggregation at the core, whereas 2D projection of protonated Lipid-5 mass distribution into the *x–y* plane (in membrane plane) for both bilayer leaflets was used to quantify Lipid-5 aggregation on the surface of the bilayer. We used the MDAnalysis^60^ Python library to estimate these quantities.

#### 2.5.6 Cholesterol Core–Surface Partitioning

The cholesterol core–surface partitioning is defined as a ratio between the number of cholesterols on the surface and at the hydrophobic region of the bilayer and calculated using the following equation:
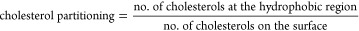
where the surface presented cholesterols are defined as those within 10 Å of the bulk solution.

#### Estimation of Lipid Clustering

Lipid clustering is performed using DBSCAN^61^ with radius and minimum number of cluster chosen to be 5 Å and 50, respectively.

## 3 Results and Discussion

### 3.1 Lipid-5 Partitioning into the Bilayer

**Figure 2:**
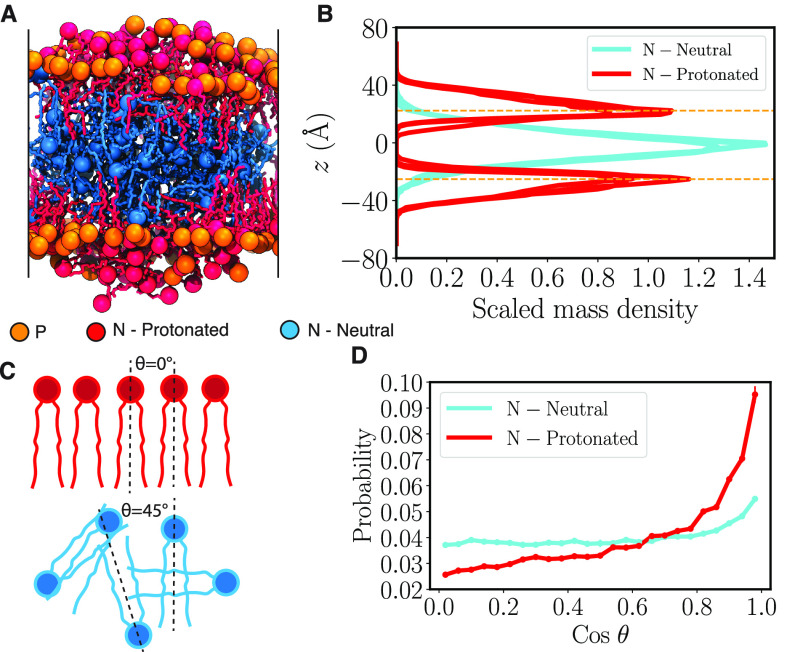
Lipid-5 partitioning into the bilayer. (A) Molecular image of the bilayer system containing 40% of Lipid-5 after 3 μs of equilibrium simulations. Protonated and neutral Lipid-5 are shown in red and cyan, respectively, with nitrogen atoms in the headgroup represented using colored spheres. Phosphorus atoms of DSPC lipids are shown with orange spheres. (B) Scaled mass density distribution of nitrogen atoms of Lipid-5, protonated (red) and neutral (cyan), calculated in the *z* direction (membrane normal). The highest value of the distribution is scaled to one. The average *z* value of phosphorus atoms of DSPC lipids in each leaflet are shown in orange horizontal dashed lines. (C) Definitions of crossing angle θ, which represents the angle between the adjoining ionizable amino lipid tails. The red lipids represent the protonated ionizable amino lipid while the blue ones represent the neutral counterparts. (D) Probability of cosine of θ for protonated (red) and neutral (cyan) Lipid-5.

In the course of the simulations performed for all the bilayer systems with different proportions of Lipid-5, we observed that protonated Lipid-5 has a tendency to stay on the surface of the bilayer (Figures 2A and 6, molecules shown in red) alongside the DSPC lipid molecules (Figures 2A and 6, molecules shown in orange). This observation was expected because the +1 charge in Lipid-5 increases the hydrophilicity of the molecule, keeping it on the surface of the bilayer and inducing interactions with water and anions. In contrast, the deprotonated Lipid-5 tends to partition into the hydrophobic core of the bilayer (Figures 2 A and 6, molecules represented in cyan), sandwiching between the upper and lower leaflets consisting of DSPC, cholesterol, and protonated Lipid-5. The lipid conformations on the surface and at the core of the bilayers, captured here, can be representative of the surface and core of LNPs containing Lipid-5, respectively. In order to estimate the relative placement of Lipid-5 molecules in the system, we calculated the scaled mass density for the titratable nitrogen atom of protonated and neutral Lipid-5 along the *z*-axis (membrane normal) using the method described in Section 2.5.1. Our observation for systems containing 40% Lipid-5 shows that protonated Lipid-5 is found to be 10 to 45 Å from the membrane center and peaking at 25 Å (Figure 2B, red lines), while the neutral Lipid-5 is found to be within 0 to 40 Å from the lipid bilayer center and peaks at the bilayer center (Figure 2B, cyan lines).

From Figure S1, we observe that the mass distributions of ionizable aminolipids for systems with 10, 20, 30, and 50% proportions of Lipid-5 are mostly similar to the ones observed for 40% Lipid-5 proportions with some noteworthy differences. For systems with low populations of Lipid-5 (10 and 20%), the mass distributions for protonated Lipid-5 are quite narrow, indicating that the bilayer surface remains relatively flat (Figure S1). However, increasing the proportion of Lipid-5 gives us a wider mass distribution for protonated Lipid-5, with 50% Lipid-5 having the widest distribution (Figure S1). This can be attributed to the lipid bilayer thickening and bulging, which we observe on the membrane surface as we increase the population of ionizable aminolipids (Figures 2A, 6, and S6). Moreover, the peak corresponding to the highest proportion of neutral Lipid-5 gets narrower at the bilayer center (Figure S1), suggesting that most of the neutral lipids lie in the hydrophobic core of the lipid bilayer. Thus, relatively speaking, increasing the proportion of Lipid-5 in a bilayer enables neutral Lipid-5 molecules to partition more to the hydrophobic core. Interestingly, at lower proportions of the ionizable lipids, the neutral ionizable lipid molecules are also on the lipid bilayer surface. This could be attributed to the hydrophilic ethanolamine polar group. Only when we have a critical amount of neutral lipid molecules is the hydrophobic core formed.

Partitioning of neutral ionizable aminolipids followed by the formation of a hydrophobic layer inside the bilayer has been observed in several earlier studies. For example, molecular dynamics simulations on lipid bilayers containing other ionizable aminolipids revealed that the neutral ionizable aminolipids formed a hydrophobic core inside the bilayer.^35,38^ Additionally, a study employing cryo-TEM, SAXS, NMR, and molecular dynamics simulations of membrane bilayers to investigate ionizable aminolipids segregation in the presence of POPC lipids suggested that neutral ionizable aminolipids at basic pH form a separate layer of hydrophobic region between the two membrane leaflets that propagates throughout the lipid bilayer, whereas the protonated lipids remain on the surface of the bilayer interacting with POPC molecules.^35,38^

In our simulations, we did not observe the formation of a complete layer containing only neutral ionizable aminolipids percolating throughout the membrane core; rather, we captured an amorphous droplet of neutral Lipid-5 in the hydrophobic core. Formation of an “amorphous core” in an empty LNP (without mRNA) with other ionizable aminolipids was observed and proposed before by Kulkarni et al. in a cryo-TEM study.^62^ Cryo-EM image of Lipid-5 containing LNPs has been observed to have a single lamellar region with an amorphous core,^24^ and we predict through our simulations that the core consists of mostly neutral Lipid-5 molecules while the former consists of DSPC, cholesterol, and Lipid-5. Although these LNPs consisted of mRNA while our simulations are not considering mRNA molecules at the moment, going forward, we are looking into molecular dynamics simulations of incorporating RNA along with the lipid bilayer system. Patel et al. also observed amorphous droplet formed with cholesterol analogues and Lipid-9 containing LNPs^17^ as well. Lipid-9 and Lipid-5 are both ethanolamine lipids so we also predict that the amorphous droplet in Patel et al. studies also consists of neutral Lipid-9 molecules.^17^ We believe the proportions of Lipid-5 relative to DSPC and cholesterol employed in our study is not enough to form a separate layer of neutral Lipid-5 at the bilayer center, and that is why we observe the formation of the amorphous oil droplet instead with Lipid-5.

In order to understand the relative conformations of the protonated ionizable aminolipids on the bilayer surface, we computed the crossing angles between the ionizable aminolipids using the method described in Section 2.5.2 and Figure 2C. The probability distribution of the cosine of the crossing angles shown in Figure 2D for the 40% lipid-5 system suggests that the protonated ionizable aminolipids are more likely to be parallel to each other. This was chosen because the probability distribution for cosine of the cross-angles is flat for randomly oriented lipid chains. The neutral ionizable aminolipids approximate a random distribution of crossing angles, suggesting that the neutral lipids are in the hydrophobic core of the lipid bilayers and have a more random orientation with each other. We also computed the probability distribution for crossing angles of Lipid-5 in other systems as well (Figure S2). For lower proportions of ionizable aminolipids in the bilayer, like 10%, Figures S1 and S2 suggest that the neutral lipids are still largely parallel to each other and probably because many of them are on the surface along with the protonated ionizable aminolipids and DSPC molecules. However, when the proportion of ionizable aminolipids reaches 30%, most of the neutral lipids move to the core of the bilayer and assume random orientations with respect to each other. Thus, there is a critical concentration of ionizable aminolipids needed to form this amorphous oil droplet like state.

### 3.2 Aggregation of Protonated Ionizable Aminolipids on the Surface of the Lipid Bilayer

Our investigations on the structure of membrane bilayers with different proportions of Lipid-5 revealed three different levels of aggregation: (1) low, (2) intermediate, and (3) high. For systems containing 10 and 20% Lipid-5, we observed that neutral ionizable aminolipids are found both on the surface and at the hydrophobic core of the bilayers, while protonated ionizable aminolipids are only on the hydrophilic surface, as shown in Figures 2B, 6, and S1. The distribution of neutral Lipid-5 molecules is likely driven by multiple competing interactions. The curvature implied by Lipid-5’s long hydrocarbon chains and very small polar headgroup suggests it should not be especially stable in a flat leaflet, but transferring a whole molecule into the hydrophobic core requires desolvating the polar ethanolamine group. The latter quantity dominates when the lipid is charged, but once the proton is removed, the desolvation penalty can be overcome.

**Figure 3:**
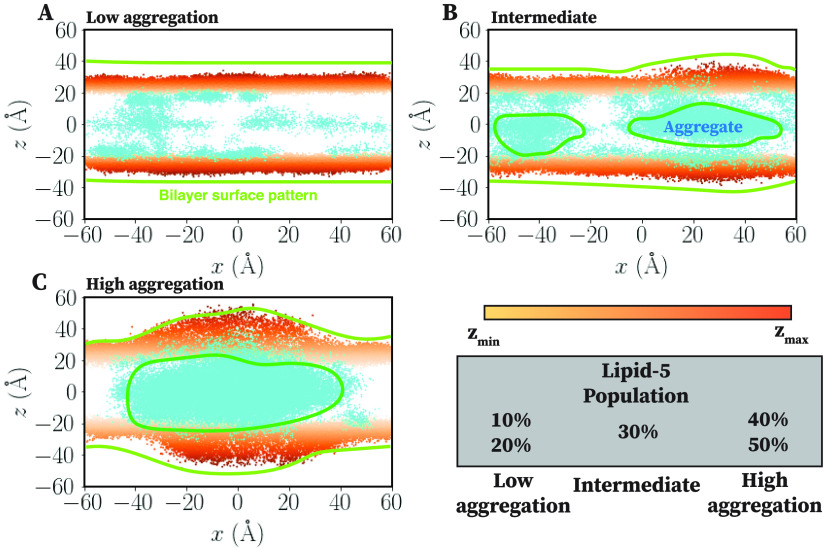
Aggregation of neutral Lipid-5 at the hydrophobic region of the bilayer. Coordinates of phosphorus atom (orange dots) of DSPC and nitrogen atom of neutral Lipid-5 (cyan dots) molecules, projected into the *x–z* plane for low aggregation, intermediate, and high aggregation states, shown in (A), (B), and (C), respectively. Membrane bilayers containing 10, 20, and 50% Lipid-5 are representative here for low aggregation, intermediate, and high aggregation states, respectively. Color spectrum of phosphorus atoms represents their positions in the *z* direction, with light and dark orange being the minimum and maximum values in one leaflet. Membrane bilayer surface pattern is highlighted using yellow lines, while the Lipid-5 clustering at the hydrophobic core is represented using cyan enclosed shapes. In low population of Lipid-5 (10 and 20%) bilayers are fallen into the low aggregation state. However, increasing the population of Lipid-5 to 30% generates the intermediate state with more aggregation of neutral Lipid-5 at the bilayer core. The high aggregation state was observed for high populations of Lipid-5 (40 and 50%), forming a single cluster and encompassing all neutral Lipid-5 at the hydrophobic core.

The cumulative spatial distribution of the titratable nitrogen atom of neutral ionizable aminolipids was projected onto the *x–z* plane along the course of the simulations in Figures 3 and S3. The first two rows of Figure S3 (simulations with 10 and 20% Lipid-5) suggest that the population of Lipid-5 in these systems in comparison to DSPC and cholesterol molecules is not enough for neutral ionizable aminolipids to aggregate between the two leaflets of the bilayer and cause bulging. Furthermore, the bilayer surface (estimated from the distribution of DSPC phosphorus atoms along the membrane normal) holds its flat shape in these two systems (Figures 3A and S3) with a thickness of 10 Å, indicating that 10 and 20% Lipid-5 populations do not cause any drastic changes in the shape of the bilayer. Figure S3 also suggests that the distribution of the neutral lipids spans the whole bilayer breadth for 10 and 20% ionizable aminolipid-containing systems. Until a critical amount of ionizable lipids is reached, the lipid bilayer remains quite flat.

**Figure 4:**
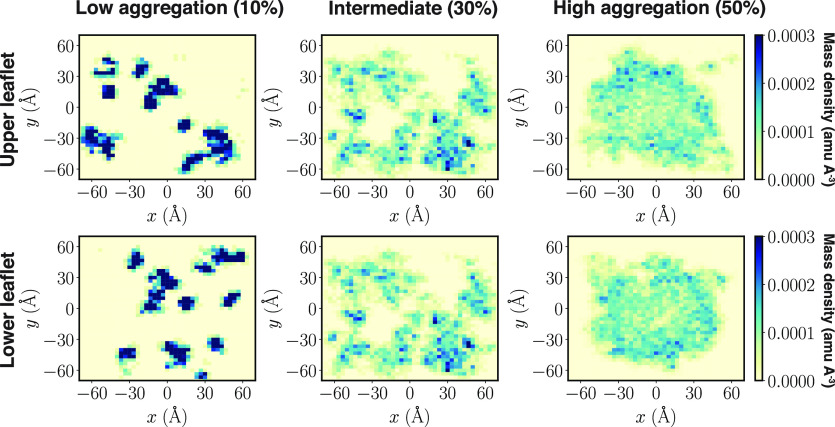
Aggregation of protonated Lipid-5 on the surface of the bilayer. Mass distribution of the nitrogen atom of protonated Lipid-5 located at the upper and lower leaflet, projected to the *x–y* plane (membrane plane). The colormap represents the mass density of protonated Lipid-5 with blue and light yellow being the minimum and maximum populations, respectively. The distributions are shown for systems with 10% (left), 30% (middle), and 50% (right) Lipid-5, representing low aggregation, intermediate, and high aggregation states, respectively.

**Figure 5:**
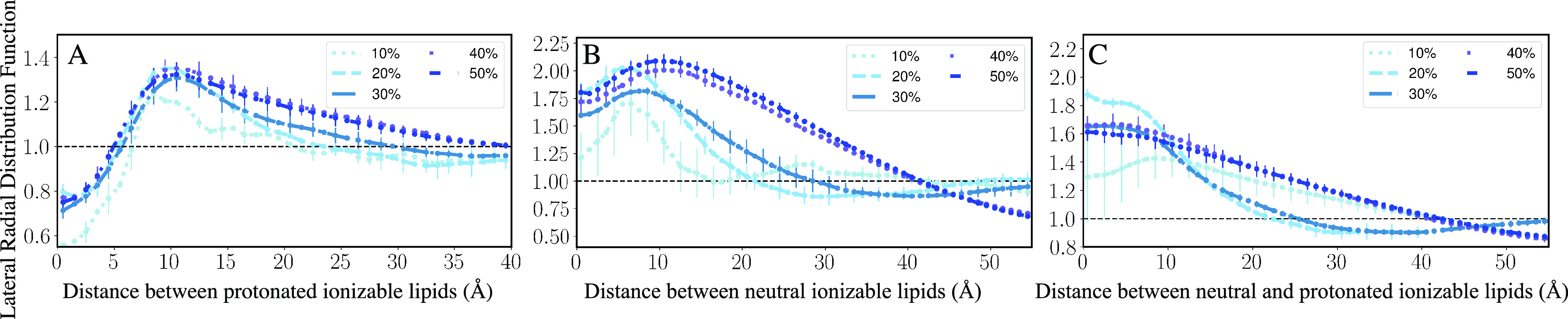
Lateral radial distribution function of (A) protonated Lipid-5 on the surface of the bilayer, (B) of neutral Lipid-5’s, and (C) of protonated and neutral Lipid-5. The radial distribution function was estimated along the *x–y* plane (membrane plane) for all the combinations of protonated and neutral lipids. The legend shows the proportion of ionizable lipids present in each line plot. The error bars show standard error while the line runs through the mean for each lipid composition. Standard error was estimated from the replicates for 10, 20, 30, 40, and 50% ionizable lipid systems. The *y* range for the plots are different because the lateral RDF values are varying depending on the different lipid species.

For protonated ionizable aminolipids, we estimated the positions of the titratable nitrogen using the method described in Section 2.5.5 and estimated the lateral radial distribution function using Section 2.5.3. We found that a lower proportion of ionizable aminolipids form small aggregates on the surface of the bilayer, as evident in Figure 4 (low aggregation). The highly dense regions in Figure 4 (low aggregation) are supported by similar patterns in Figure S4 for the other replicates of the 10 and 20% ionizable aminolipid systems. This observation was also supported by a peak in the lateral radial distribution function shown in Figure 5A for 10% ionizable aminolipids with the distance between the centroids of the lipids of about 10 Å.

Increasing the proportion of Lipid-5 from 10 to 30% changes the behavior of the neutral ionizable aminolipid molecules as well; the neutral Lipid-5 molecules aggregate more in the hydrophobic core of the lipid bilayer, forming small aggregates (Figure 3B) sandwiched between the two lipid layers. This in turn induces some structural changes to the surface of the bilayer, thickening and bulging the bilayer (Figures 3B and S3). The broadening of the lateral radial distribution curves for neutral lipids in Figure 5B also suggests that larger aggregates are formed as the number of ionizable lipids increases. The presence of significant density at zero distance is due to the presence of a nonlamellar aggregate in the center of membrane; the distance calculation is performed in the *x*–*y* plane, so the presence of lipids “below” the leaflet shows up as density at small distances. As the fraction of Lipid-5 increases, the first peak in the lateral RDF shown in Figure 5C for neutral and protonated Lipid-5 flattens, showing that the two species do not tend to colocalize. At higher proportions of Lipid-5 (40 and 50%), we observed a high aggregation of neutral Lipid-5 in the hydrophobic core of the bilayers, forming a single large aggregate (Figures 3C and S3).

**Figure 6:**
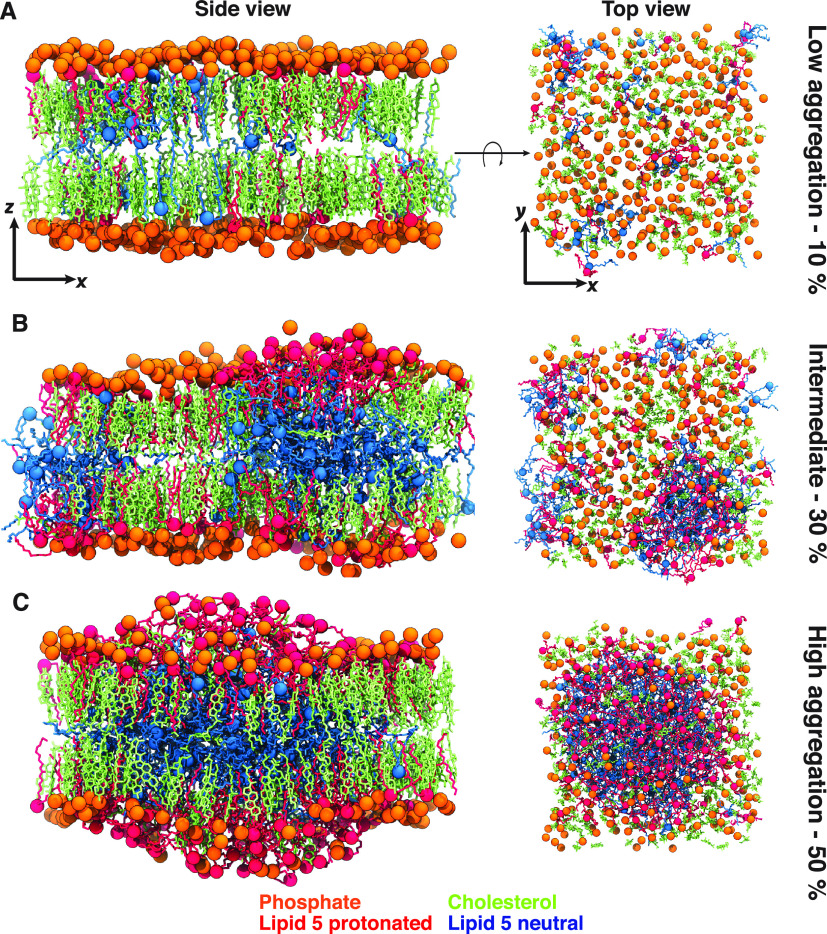
Molecular images of membrane bilayers for different compositions. Molecular images captured from one of the simulated replicas of systems containing 10, 30, and 50% Lipid-5, which are representative for low aggregation (A), intermediate (B), and high aggregation (C) states, respectively. Each image was taken from the last snapshot of each trajectory after 3 μs of simulations, with side (*x–z* plane) and top (*x–y* plane) views represented in left and right panels, respectively. Protonated and neutral Lipid-5 are shown in red and cyan, respectively, with the nitrogen atom in each ionizable lipid highlighted in colored spheres. The phosphorus atom of each DSPC is shown in orange. Cholesterol molecules are represented in green.

Furthermore, in 30% Lipid-5 systems, the protonated ionizable aminolipids form more numerous and larger aggregates on the surface of the bilayer in comparison to the low aggregation state (Figures 4, S4, and S5). This is also evident in the lateral radial distribution function (RDF) for the protonated ionizable aminolipids plotted in Figure 5A for 30% ionizable aminolipid systems. With increasing proportions of ionizable aminolipids in the system, the RDF peak broadens, and it happens in all cases when we reach 30% ionizable lipids, suggesting large aggregate formation as well as a transition from smaller aggregates to larger aggregates.

For previously published data on LNPs, cryo-EM images indicated that both protonated and neutral lipids are present in the core of the lipid nanoparticles.^63^ In addition, they claimed protonated ionizable aminolipids along with DSPC and cholesterol form concentric bilayers with the RNA trapped between two consecutive bilayers.^63^ We observed that protonated Lipid-5 molecules tend to form a single cluster on the surface of the bilayer (Figures 4, S4, and S5) which could be the first layer inside the core of the LNP as observed in the cryo-EM image.^24^ These results suggest that lipid composition can modulate LNP structure and show that unless we have a certain amount of neutral Lipid-5, we cannot form a stable core of the lipid nanoparticle. However, the simulation results we observe may depend on system size and can be sensitive to the relative population of Lipid-5 with respect to other components in the bilayer and the total number of lipids forming the membrane bilayer.

### 3.3 Changing Membrane Properties at Different Proportions of Lipid-5

**Figure 7:**
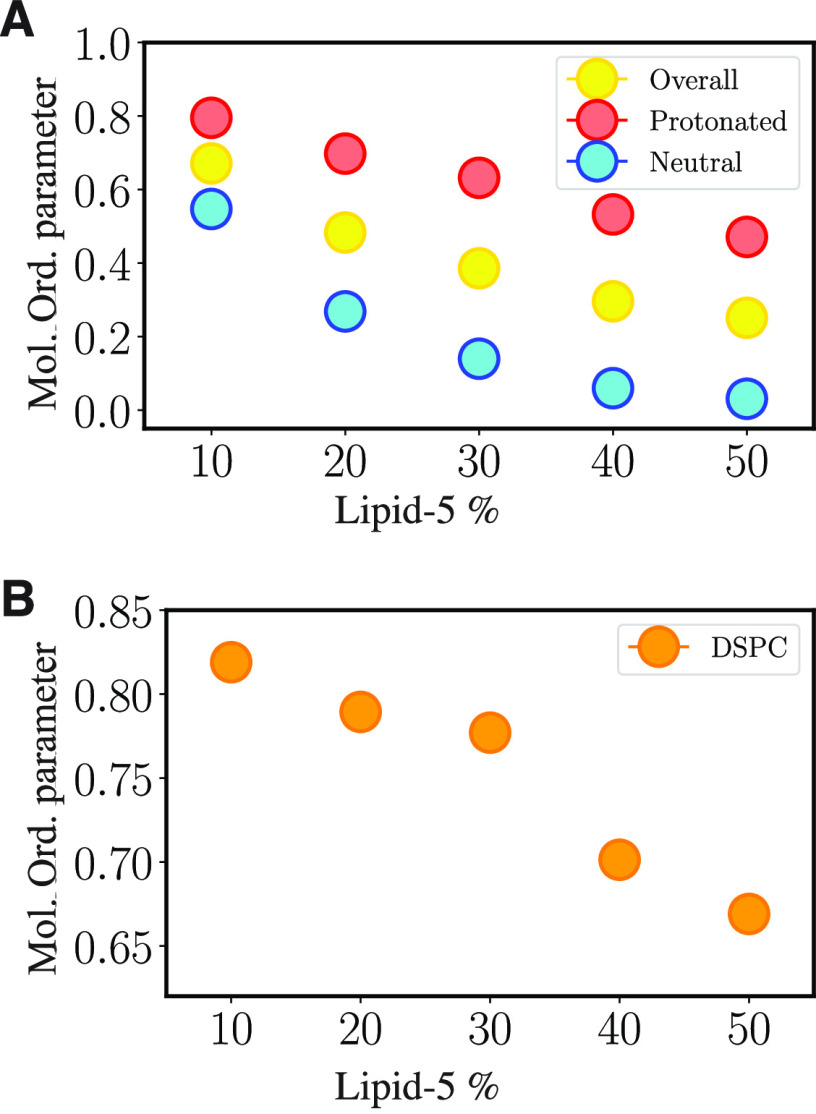
Order parameter of different bilayer components. (A) Overall, protonated, and neutral Lipid-5 order parameters for bilayer systems with different proportion of Lipid-5 are shown in yellow, red, and cyan, respectively. The order parameter of Lipid-5 decreases as its populations increases in the bilayer. (B) DSPC order parameter for system with different proportions of Lipid-5. Order parameter of DSPC decreases at the high aggregation states (40 and 50% of Lipid-5).

Structural transitions captured at different proportions of ionizable aminolipids affect several surface properties of the membrane bilayer systems simulated in this study. The lipid order parameter (defined in Section 2.5.4) indicates that larger tilt angles of the lipid on the lipid bilayer surface correlate with lower average modified order parameters. Increasing the proportions of Lipid-5 decreases the order of both protonated and neutral Lipid-5. The highest modified order parameter for protonated Lipid-5 is 0.80, captured for systems containing 10% ionizable aminolipids, whereas the lowest value is 0.47, corresponding to systems containing 50% of ionizable aminolipids (Figure 7A red dots and Table S2). Overall, the neutral Lipid-5 molecules tend to have smaller order parameters in comparison to their protonated counterparts (Figure 7A and Table S2). The modified order parameter for neutral ionizable aminolipid is 0.55, which belongs to systems containing 10% of Lipid-5, whereas the lowest value is 0.03 and is measured for systems with 50% Lipid-5 (Figure 7A, cyan dots, and Table S2). This could be also attributed to the random orientation of the neutral Lipid-5 in the hydrophobic core formed at higher proportions of the ionizable aminolipid systems. The modified order parameter is a measure of how lipids with varying structures impact each other. The main reasons for the modified order parameter to be lowered for DSPC molecules and protonated Lipid-5 would be: (a) The Lipid-5 small polar group with branched tails impacts how ionizable aminolipids pack with each other and with DSPC molecules on the surface. (b) Formation of the neutral Lipid-5 aggregate causes bulging of the lipid bilayer, which can cause accumulation of protonated ionizable aminolipids on the surface to release the tension caused by the aggregates. This observation was expected because the geometry and curvature of the bilayer surface change more drastically locally wherever the neutral lipids accumulate.

Similar to that of Lipid-5, the order parameter for DSPC molecules decreases as the bilayers become more populated with ionizable aminolipids. The maximum and minimum value of DSPC order parameters are 0.82 and 0.67, which are captured for systems containing 10 and 50% ionizable aminolipids, respectively (Figure 7B and Table S2). Although the trend of DSPC and Lipid-5 order parameters are similar (the higher the proportion of ionizable aminolipids, the smaller the order parameter), the DSPC order parameter decreases drastically (relatively speaking) as the bilayer transitions to the high aggregation state. This can be attributed to the fact that the modified order parameter measurement uses a global membrane normal instead of the local leaflet normal as the leaflet curves to accommodate the neutral lipids. Hence, we might be observing this change in lipid order because we are not estimating local order parameters. The DSPC and cholesterol molecules also seem to be segregating and appear to occupy the areas on the bilayer surface less populated with protonated Lipid-5 molecules as evident in Figures 6B and 6C. This might also explain why the order parameters vary with Lipid-5 proportions.

**Figure 8:**
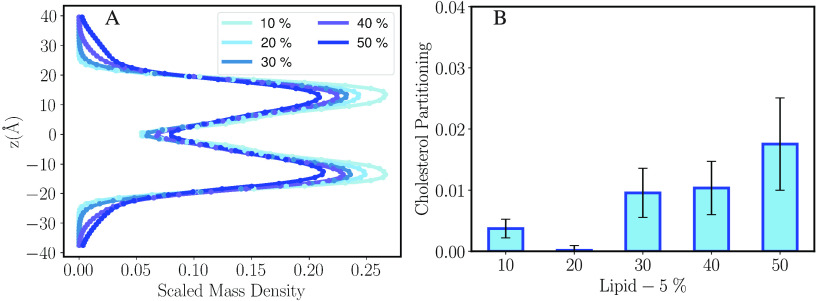
(A) Scaled mass density distribution of cholesterol calculated in the *z* direction (membrane normal). The highest value of the distribution is scaled to one. The error bars are small so they appear as dots here. (B) Cholesterol core–surface partitioning estimation for different proportions of Lipid-5.

Previous MD simulations on LNP assembly and lipid composition of LNP surface and core indicated the existence of cholesterol molecules in the core.^38,62^ Inspired by this result, we calculated the mass density of cholesterol molecules along the membrane normal as shown in Figure 8A. We observe that at low Lipid-5 density, the cholesterol is almost exclusively found in the leaflets, but at higher densities some of the cholesterol partitions into the interior. In addition, we also estimated the cholesterol core–surface partition coefficient value as defined in Section 2.5.6 (Figure 8B) to monitor the ratio of cholesterol molecules moving to the core of our lipid bilayer systems in different proportions of ionizable aminolipids. Cholesterol core–surface partitioning for systems containing 10 and 20% ionizable lipids is less than 0.01, indicating that all of the cholesterol molecules in these systems remain on the surface of the bilayer in conjunction with protonated Lipid-5 and DSPC molecules (Figure 8B and Table S3). As the proportion of Lipid-5 increases, we observe more cholesterol partitioning to the hydrophobic core of the bilayer (Figures 8B and S8, Table S3). The cholesterol core–surface partition coefficient was measured for systems containing 50% with an average of 0.0223 (Figure 8B and Table S3). Cholesterol partitioning to the hydrophobic core of bilayers containing ionizable aminolipids in our simulations was expected as cholesterol is mostly hydrophobic except for the small polar head, resulting in a modest penalty to partition into the core. Several groups have reported this phenomenon in lipid nanoparticles, but they have not been able to find relationship between cholesterol and increasing proportion of ionizable lipids.^64,65^ Trollman et al. used all-atom molecular dynamics to demonstrate the partitioning of cholesterol into the membrane interior at high pH. Our results are consistent with theirs, in that the presence of a significant population of neutral Lipid-5 (supplied in our case by a change in composition rather than pH) stabilizes the aggregation of nonlamellar lipids in the membrane interior.^66^ Here we have shown that for Lipid-5 there is some correlation between increasing the proportion of neutral lipids and cholesterol partitioning to the core. Because cholesterol content is known to modulate membrane surface properties, this could explain why the modified order parameters of the DSPC molecules are impacted as the ionizable aminolipid content changes.^67,68^

In our simulations, we have observed that increasing the neutral lipid proportion induces the amorphous droplet formation in the hydrophobic core of the lipid bilayer. This causes a bulging of the membrane bilayer surface. Protonated Lipid-5 molecules which have a small polar group (ammonium ion) and highly branched tails occupy the surface of the bilayer, which contains the bulge formed by the neutral ionizable aminolipids. This phenomenon causes the overall lipid bilayer to have a lower order parameter and increases the proportion of cholesterol that accumulates in the hydrophobic core of the lipid bilayer.

We have parametrized Lipid-5 using GAFF2 parameters using an antechamber which has low accuracy.^48^ Lipids parameters are usually optimized to experiments like surface tension measurements, neutron scattering, and NMR data.^69^ Although we have not used these methods, our simulations have some intrinsic errors, but we can explain some of our observations with the experiments run for LNPs containing Lipid-5.^24^

Lipid nanoparticles have a complex structure, and their degree of lamellar phase depends on the chemical structure of the ionizable aminolipid.^24^ Our lipid bilayers consisting of varying proportions of the ionizable lipids can be representative models for the different lamellar phases found on the surface as well as inside the LNP. The hydrophobic core consisting of Lipid-5 molecules formed in the high aggregation state in our simulations can be a representation of the electron dense core observed in previous investigations with some of them having ionizable lipids that are similar in chemical structure to Lipid-5.^17,24,70^ Thus, our simulation protocol and the results mentioned here can serve as templates to build structure activity relationships for new ionizable aminolipids.

## 4 Conclusion

In summary, we developed a computational method to incorporate novel ionizable aminolipids into a lipid bilayer for molecular dynamics simulations. We performed long-time scale MD simulations on ternary bilayer systems containing Lipid-5 (an ionizable aminolipid), DSPC, and cholesterol molecules for 3 μs (with an aggregate simulation time of 45 μs for all the systems). The lipid bilayer systems maintain the same mole fraction of cholesterol (40%), whereas DSPC and Lipid-5 proportions vary from 10 to 50%. The lipid bilayers studied here each contain the same proportion of neutral and protonated Lipid-5, resembling the microenvironment of the LNP surface. The simulations describe how the structure and distribution of the different lipid species vary with composition. In addition, our results suggest that as the neutral ionizable aminolipid proportion increases and reaches a critical amount in the lipid bilayer, the membrane transitions to a high aggregation state (40 and 50% Lipid-5 systems), where we observe a hydrophobic core formed by neutral Lipid-5 and cholesterol molecules which is crucial for imparting stability to the LNP. Characterization of membrane bilayers simulated here shows that populating the lipid bilayer with more Lipid-5 decreases the modified order parameter of the lipid molecules, generating a disordered membrane which can ensure mRNA encapsulation. The curvature change induced by the hydrophobic core causes a significant rearrangement of the lipids on the surface where we observe protonated Lipid-5 aggregating due to the more inverted cone shape of the lipids, facilitating occupation on a more curved surface. The formation of the hydrophobic core in high aggregation systems also causes cholesterol to partition to the core of the LNPs which is also responsible for the observed changes on the lipid bilayer surface. Hence our work provides key insights into the balance imparted by the ionizable aminolipids to the LNP, and their amount and protonation states impact the LNP surface directly. Overall, the finding of this study can be used in deciphering a suitable lipid composition for LNPs in mRNA therapeutics applications as well as provide a deeper understanding of lipid–lipid interactions essential for LNP formation and stability.

## Data Availability

All the analysis scripts as well as the insertion algorithm are available at https://github.com/modernatx/ionizable_amino_lipids_distribution.

## Supporting Information Available

Table S1: Five simulated systems containing the same proportion of cholesterol (40%) and different proportions of DSPC (50 to 10%) and Lipid-5 (10 to 50%); Figure S1: Lipid-5 scaled mass density in different lipid bilayer systems; Figure S2: Lipid-5 orientation angle with respect to membrane normal for different lipid bilayer systems; Figure S3: Neutral Lipid-5 aggregation at the hydrophobic core of the bilayer for different lipid bilayer systems; Figure S4: Protonated Lipid-5 aggregation at the upper leaflet for five different lipid bilayer systems; Figure S5: Protonated Lipid-5 aggregation at the lower leaflet for five different lipid bilayer systems; Figure S6: Molecular images of the membrane bilayer systems viewed from the side for five different lipid bilayer systems; Figure S7: Molecular images of the membrane bilayer systems viewed from the top for five different lipid bilayer systems; Table S3: mean (μ) and standard deviation (σ) of cholesterol partition coefficient for all lipid bilayer systems; Figure S8: Time series of bilayer characteristics in all five different systems with different proportions of Lipid-5 (PDF)
