# Supplementary material for: Ionizable Amino Lipids Distribution and Effects on DSPC/Cholesterol Membranes: Implications for Lipid Nanoparticle Structure
Source: J Phys Chem B. 2023 Jul 27;127(31):6928–39. doi: 10.1021/acs.jpcb.3c01296 (PMC10424244; doi:10.1021/acs.jpcb.3c01296)
Supplement: Supplementary file 1 — jp3c01296_si_001.pdf [file jp3c01296_si_001.pdf]

Ionizable Amino Lipids Distribution and Effects on DSPC/cholesterol Membranes: Implications for Lipid Nanoparticle Structure

Sepehr Dehghani-Ghahnaviyeh[1,2], Michael Smith[1], Yan Xia[1], Athanasios Dousis[1], Alan Grossfield[3], and Sreyoshi Sur[1,*]

[1]Moderna, Inc., Cambridge, Massachusetts, United States

[2]Theoretical and Computational Biophysics Group, NIH Center for Macromolecular Modeling and Bioinformatics, Beckman Institute for Advanced Science and Technology, Department of Biochemistry, and Center for Biophysics and Quantitative Biology, University of Illinois at Urbana-Champaign, Urbana, Illinois, United States of America

[3]Department of Biochemistry and Biophysics, University of Rochester Medical Center, Rochester, NY, United States of America

[*]Corresponding author: Sreyoshi Sur, sreyoshi.sur@modernatx.com

Systems	Lipid-5 %	DSPC %	Cholesterol %	of replicates	Simulation time per replicate	Production run per replicate
1	10	50	40	3	3 μs	1 μs
2	20	40	40	3	3 μs	1 μs
3	30	30	40	3	3 μs	1 μs
4	40	20	40	3	3 μs	1 μs
5	50	10	40	3	3 μs	1 μs

Table S1: **Simulated systems.** Five simulated systems contain the same proportion of cholesterol (40 %), and different proportions of DSPC (50 to 10 %) and Lipid-5 (10 to 50 %). Each system was replicated three times and simulated 3 μs per replica, resulting in 9 μs of simulations per system, and cumulative simulation time of 45 μs for all systems.

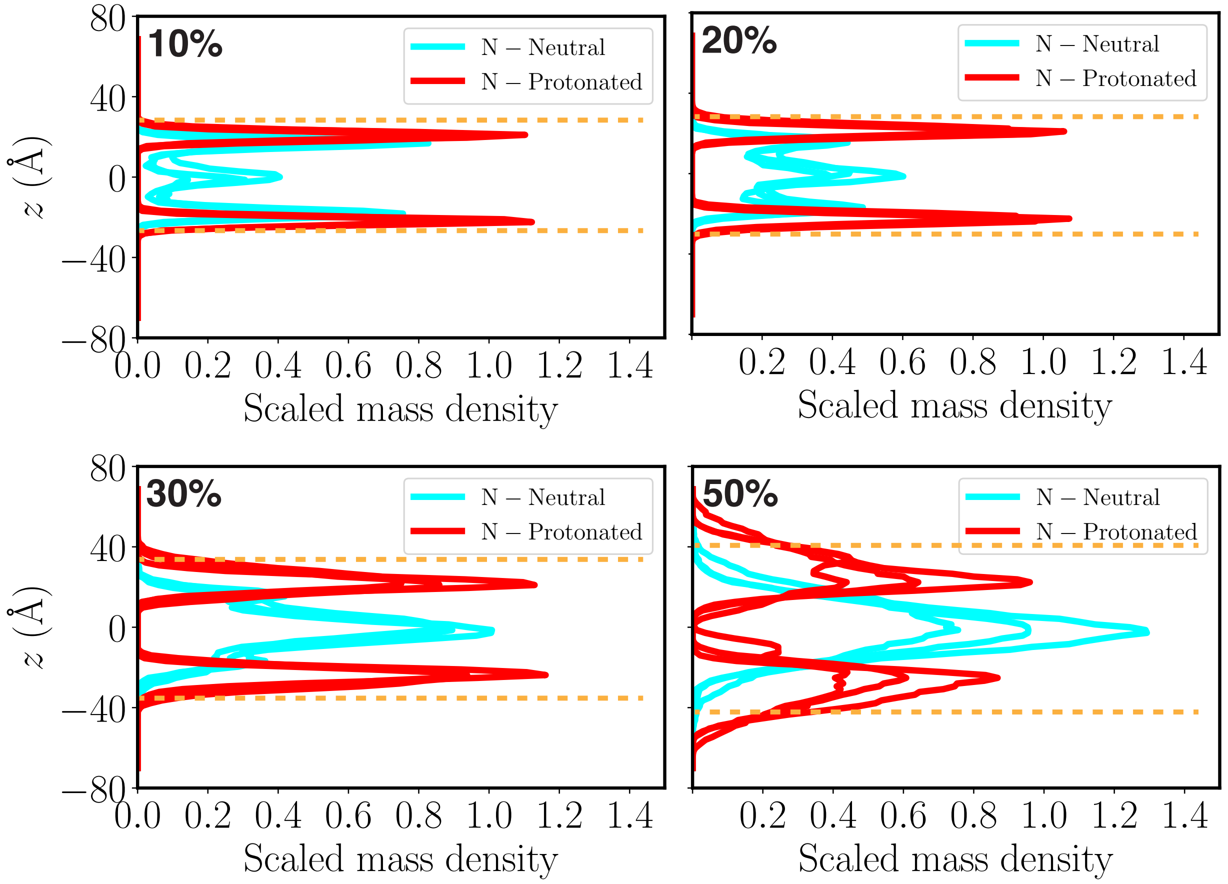

Figure S1: **Lipid-5 scaled mass density in different bilayer systems**. Scaled mass density of the titrable nitrogen of neutral (cyan) and protonated (red) Lipid-5 with respect to z (membrane normal) for systems with 10, 20, 30, and 50 % of Lipid-5. The maximum count in each distribution is scaled to one. The average z position of phospholipid headgroup in each leaflet is shown in orange dashed lines.

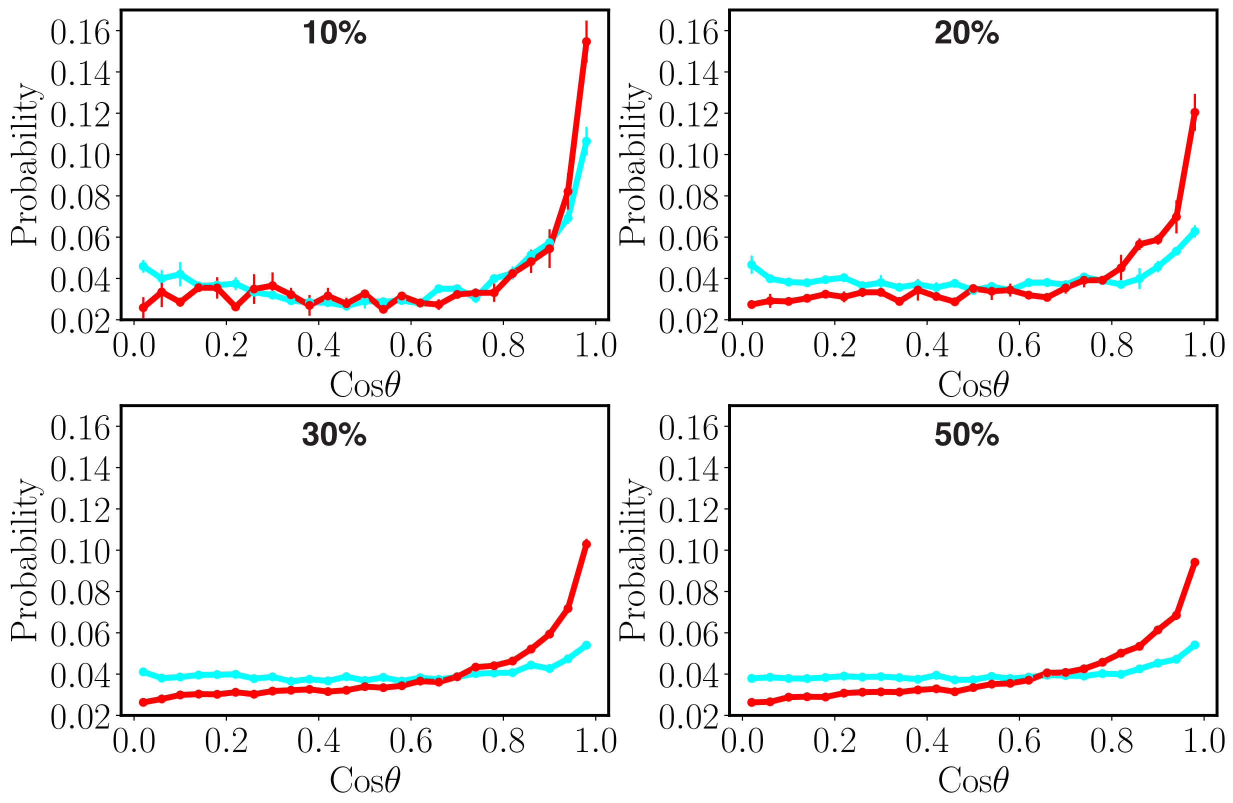

Figure S2: **Lipid-5 orientation angle with respect to membrane normal**. PDF of orientation angle. θ, of neutral and protonated ionizable lipids with respect to membrane normal for systems with 10, 20, 30, and 50 % Lipid-5, shown in cyan and red, respectively. As Lipid-5 population increases, more neutral ionizable lipids aggregate at the hydrophobic core of the bilayer, resulting in a more perpendicular orientation of neutral Lipid-5. Protonated Lipid-5 peak around 20 and 160 $^\circ$, corresponding to their orientation in upper and lower leaflets, respectively.

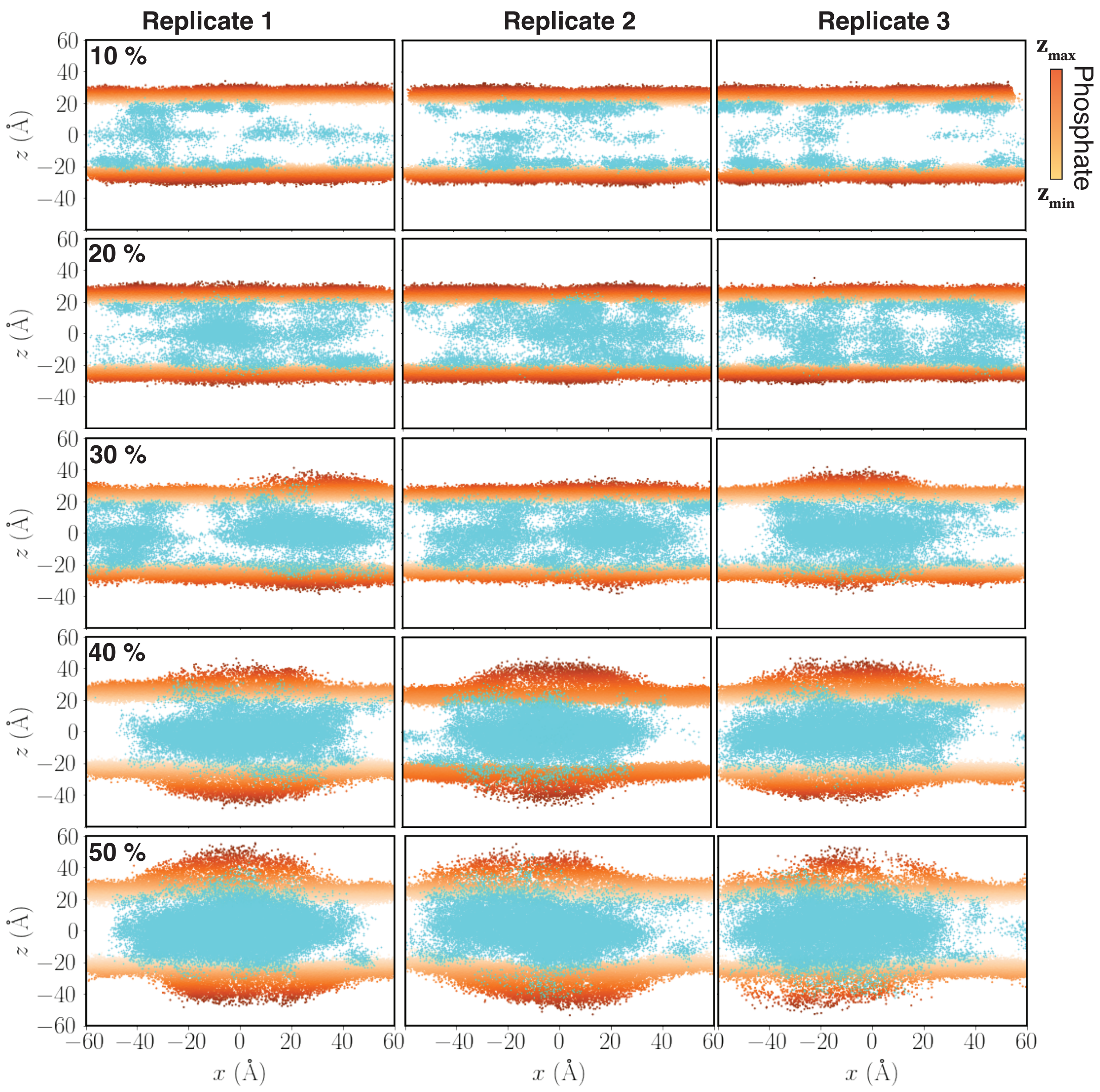

Figure S3: **Neutral Lipid-5 aggregation at the hydrophobic core of the bilayer**. Coordinates of phosphorus (orange) and nitrogen (cyan) atoms of DSPC and neutral ionizable lipids, respectively, in *x*-*z* plane for systems with different populations of Lipid-5. Phosphorus atoms are colored in each leaflet based on their *z* distance with respect to membrane center, with light and dark orange being the smallest and largest distance, respectively. High populations of Lipid-5 (40 and 50 % in this case) results in aggregation of neutral Lipid-5 at the hydrophobic core of the bilayer, forming a single cluster.

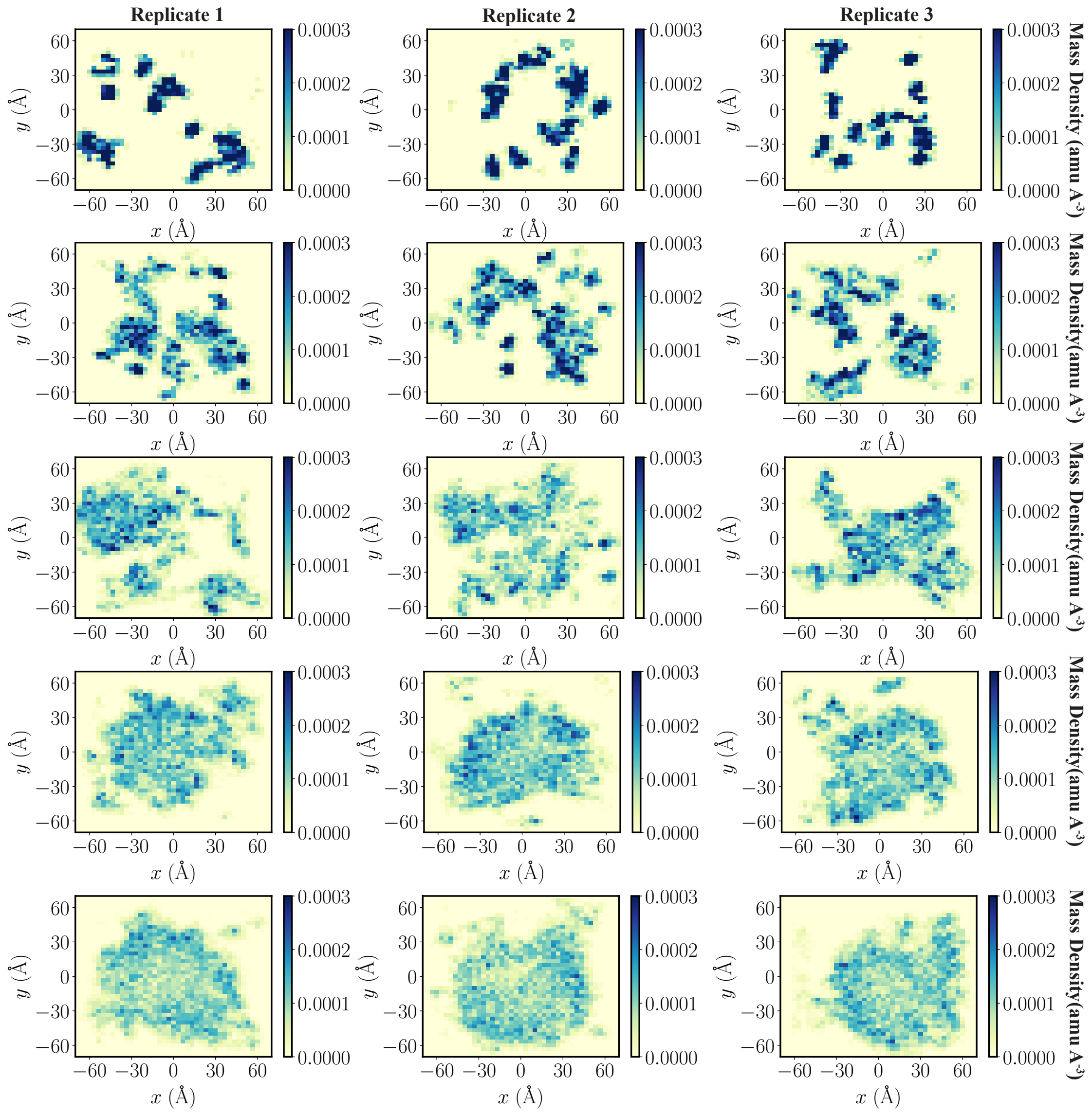

Figure S4: **Protonated Lipid-5 aggregation at the upper leaflet of the bilayer**. Top view (*x*-*y* plane) of mass distribution of the nitrogen atom of protonated Lipid-5, located at the upper leaflet for systems with different populations of Lipid-5. Protonated Lipid-5 tend to aggregate at high population of Lipid-5 (40 and 50 % in this case).

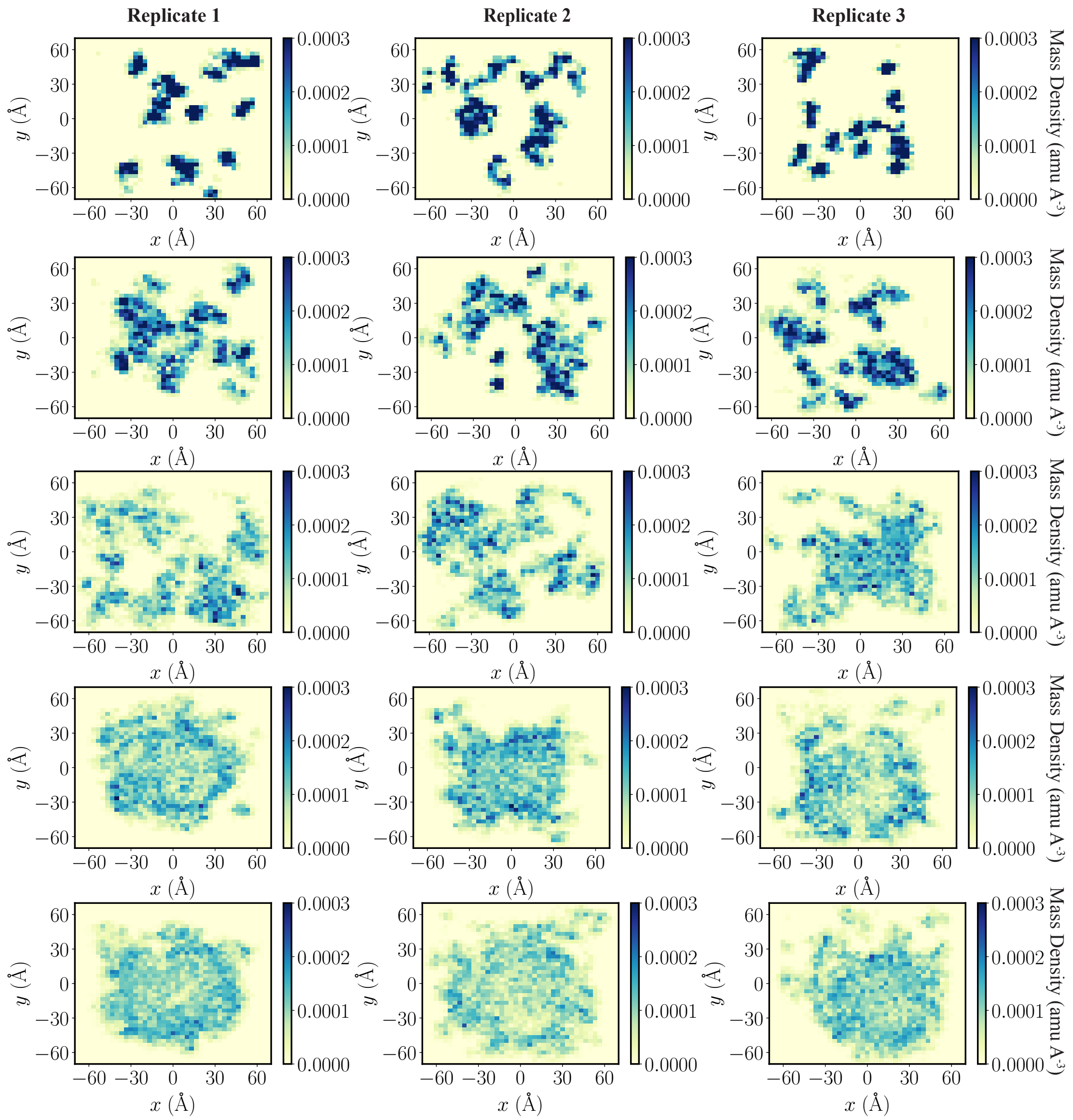

Figure S5: **Protonated Lipid-5 aggregation at the lower leaflet of the bilayer**. Bottom view (*x*-*y* plane) of mass distribution of the nitrogen atom of protonated Lipid-5 at the lower leaflet.

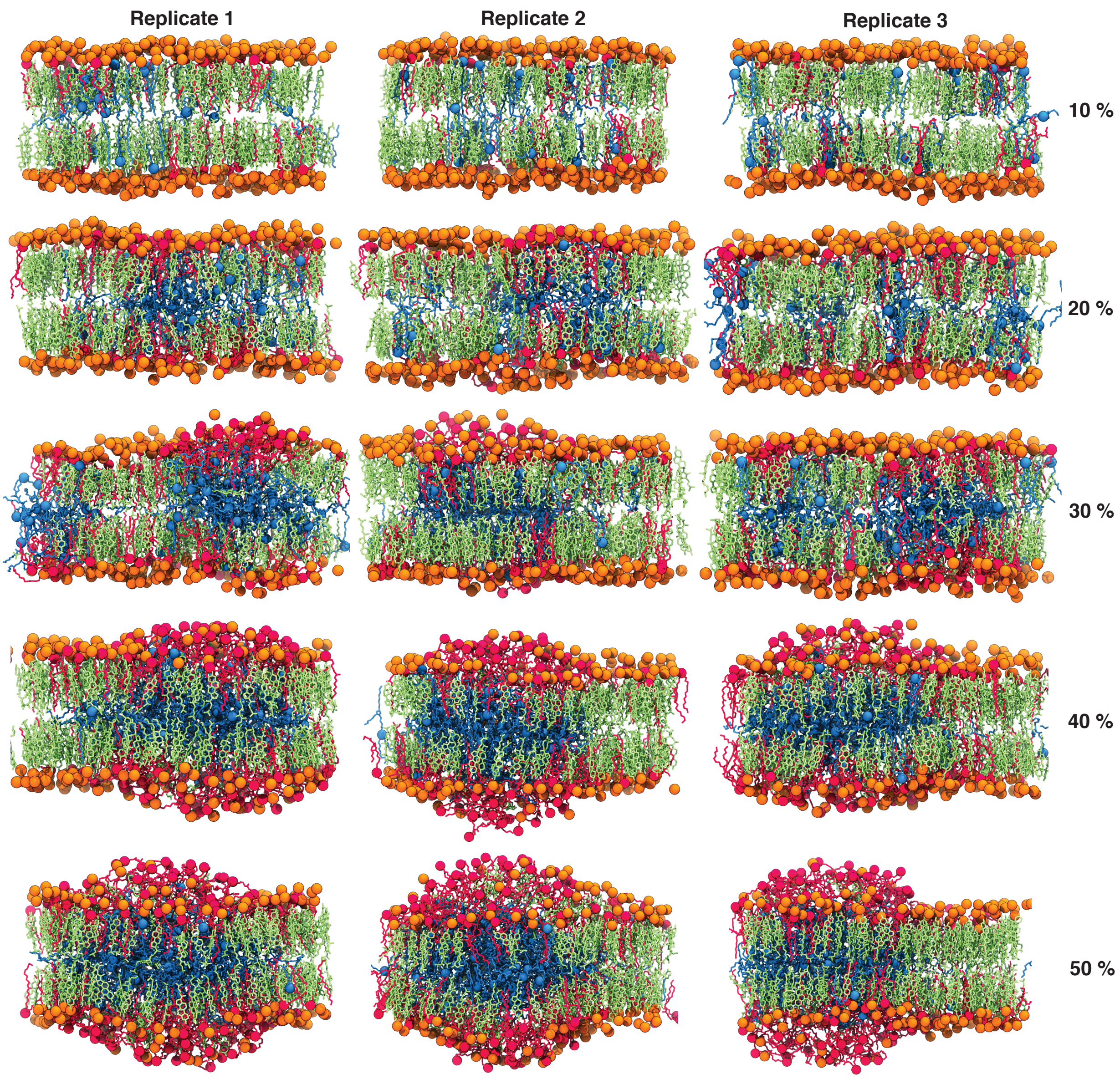

Figure S6: **Molecular images of the membrane bilayer systems viewed from the side**. Molecular images of all the simulated systems captured from the last snapshot of each trajectory (after 3 μs of simulations). Columns and rows are categorized based on replicas and proportion of Lipid-5, respectively. Protonated and neutral Lipid-5 are shown in red and cyan, respectively, with nitrogen atom of each ionizable lipid represented in colored sphere. Phosphorus atoms of DSPC molecules are shown in orange spheres. Cholesterol molecules are represented in green. Increasing the population of Lipid-5 generates new structural states for the bilayer, resulting in aggregation of protonated and neutral Lipid-5 on the surface and at the core of the bilayer, respectively.

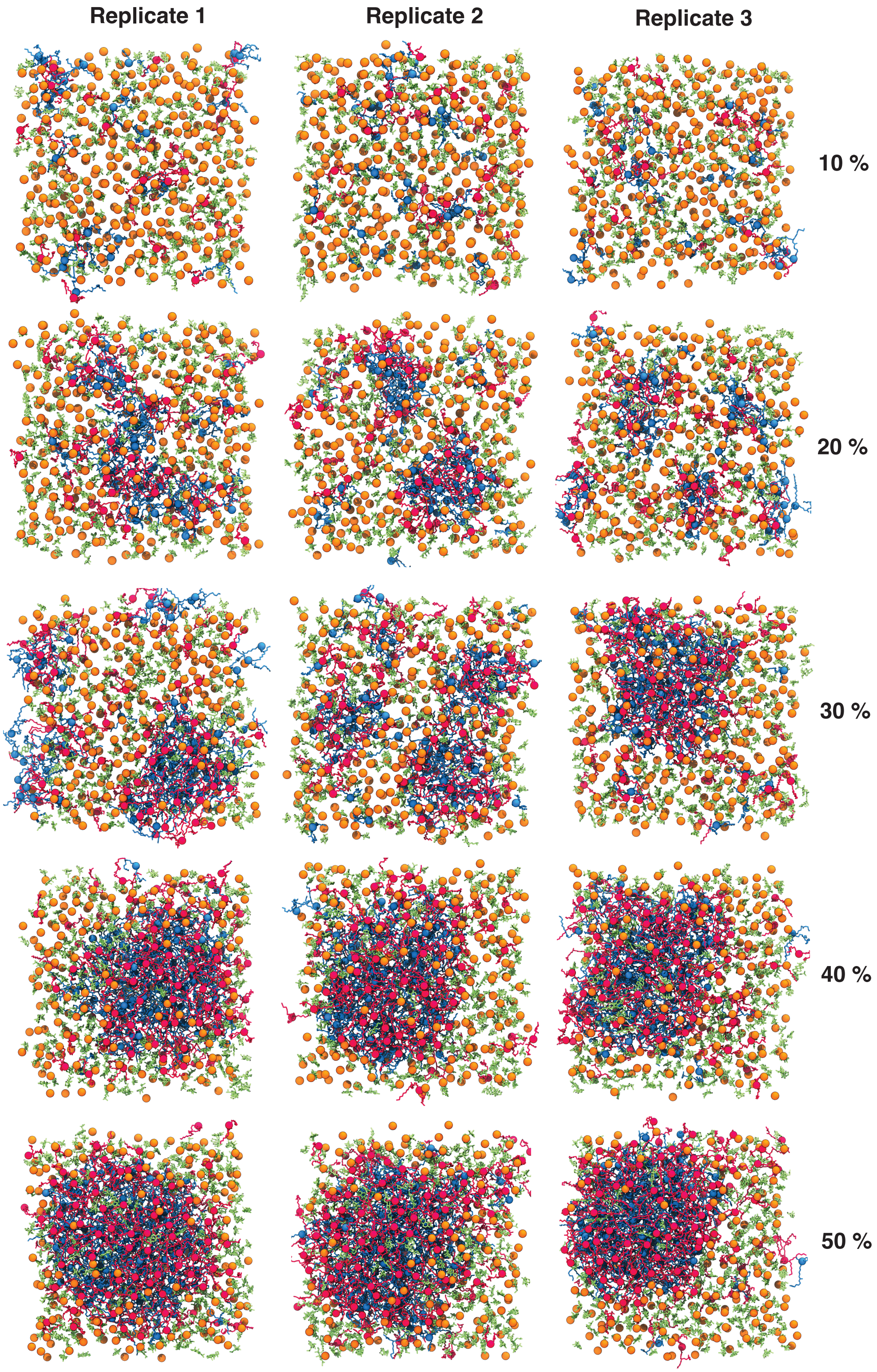

Figure S7: **Molecular images of the membrane bilayer systems viewed from the top**. Molecular images of all the simulated systems taken from each trajectory after 3 μs of simulations. Columns and rows are classified based on replicas and proportion of Lipid-5, respectively. Protonated and neutral Lipid-5 are shown in red and cyan, respectively, with nitrogen atom of each ionizable lipid highlighted in colored sphere. Orange spheres represent the phosphorus atoms of DSPC molecules. Cholesterol molecules are shown in green.

Systems	Lipid-5 %	Protonated Lipid-5	Neutral Lipid-5	Lipid-5	DSPC
1	10	0.795	0.547	0.671	0.819
2	20	0.697	0.268	0.483	0.789
3	30	0.631	0.139	0.385	0.777
4	40	0.532	0.059	0.295	0.701
5	50	0.471	0.030	0.251	0.669

Table S2: **Order parameter of Lipid-5 and DSPC molecules.** Mean and standard deviation of order parameters, calculated for neutral and protonated Lipid-5 as well as DSPC molecules. The order parameter of different lipid components decrease, as bilayers get more populated with Lipid-5.

Systems	Lipid-5 %	Partitioning coefficient (μ)	Partitioning coefficient (σ)
1	10	0.0013	0.0019
2	20	0.0000	0.0000
3	30	0.0028	0.0039
4	40	0.0095	0.0078
5	50	0.0223	0.0039

Table S3: **Mean (μ) and standard deviation (σ) of cholesterol partition coefficient** Cholesterol partition coefficient increases as bilayers get more populated with Lipid-5.

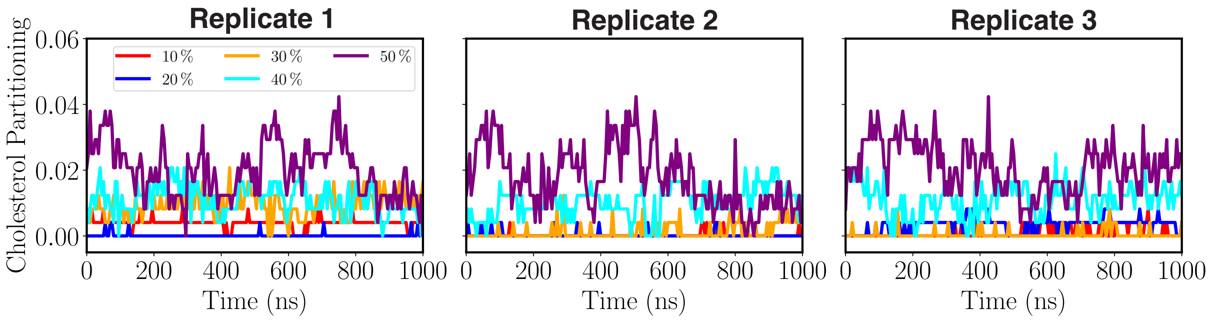

Figure S8: **Time series of bilayer characteristics in systems with different proportions of Lipid-5**. Cholesterol partitioning, calculated for last 1 μs of all simulated systems with first, second, and third replicas, represented in left, middle, and right panels, respectively.
